# Cat Dilemma: Too Protected To Escape Trophy Hunting?

**DOI:** 10.1371/journal.pone.0022424

**Published:** 2011-07-27

**Authors:** Lucille Palazy, Christophe Bonenfant, Jean-Michel Gaillard, Franck Courchamp

**Affiliations:** 1 Écologie, Systématique et Évolution, UMR-CNRS 8079, Univ Paris-Sud, Orsay, France; 2 Biométrie et Biologie Évolutive, UMR-CNRS 5558, Univ Lyon 1, Villeurbanne, France; Université Pierre et Marie Curie, France

## Abstract

Trophy hunting is one of the most controversial issues in the field of biodiversity conservation. In particular, proponents and opponents debate fiercely over whether it poses a threat to hunted populations. Here, we show that trophy hunting constitutes a greater menace to threatened species than previously realized. Because humans value rarity, targeted species that are threatened are likely to be disproportionately hunted, thereby becoming even more vulnerable, which could eventually push them to extinction. With the ten felid species currently hunted for their trophies, we present evidence that (1) the number of killed individuals increases with time, in several cases exponentially, despite population declines, (2) the price of trophies is strongly dependent on species protection status, (3) changes of protection status coincide with counter-intuitive changes of hunting pressures: protection intensification with augmented hunting effort, and protection relaxation with lower effort. This suggests an over-exploitation of trophy-hunted felids and the necessity of a better quota system coupled with reconsidered protection methods.

## Introduction

Trophy hunting is one of the most controversial issues in the field of biodiversity conservation, with fierce debates over whether it poses a threat to hunted populations [1], [2], [3]. Proponents of this multi-billion dollar industry highlight the enormous income it can generate for biodiversity conservation at the cost of a few harvested individuals of target species [1], [2], [3], [4]. They also argue that hunters are frequently instrumental in protecting hunted species by both protecting habitat and preventing poaching [3]. Opponents counter that hunting is inherently unethical and that the selective culling of individuals can have population consequences, as has been shown in antelopes, elephants, lions and bears [5], [6], [7], [8], [9]. They also claim that the high fees generated by trophy hunting lead to difficulties to control corruption in countries with high levels of poverty, and that trophy hunting is less economically profitable than photographic tourism. Opponents to trophy hunting point out as well that even the most threatened species are potential targets for trophy hunters and, in many cases, quotas are inappropriately designed or not respected [2], [10].

There is evidence to support both sides of the argument: trophy hunting has successfully been used to help some declining populations to recover. For example, a trophy-hunting based conservation program in Pakistan helped to stop the decline of two endangered Himalayan sheep and goat species [11]. Conversely, recent studies have pointed out the need to consider trophy hunting as a threat to species conservation. Notably, trophy hunting of African lions, one of the most charismatic species, is sometimes ill-managed and could be implicated in the decline of populations [12]. Similarly, as demonstrated in ungulates, rarity *per se* can be responsible for a disproportionate attractiveness of the species among trophy hunters [13], [14].

In this crucial but unsolved conundrum for the conservation of charismatic mammals, we discovered that hunter's selection criteria seems influenced by threat status: hunters could prefer species that are highly threatened. We tested it with the ten felid species that are legally hunted for their trophies and listed in the Convention on International Trade in Endangered Species of Wild Fauna and Flora (CITES).

## Results

**Figure 1:**
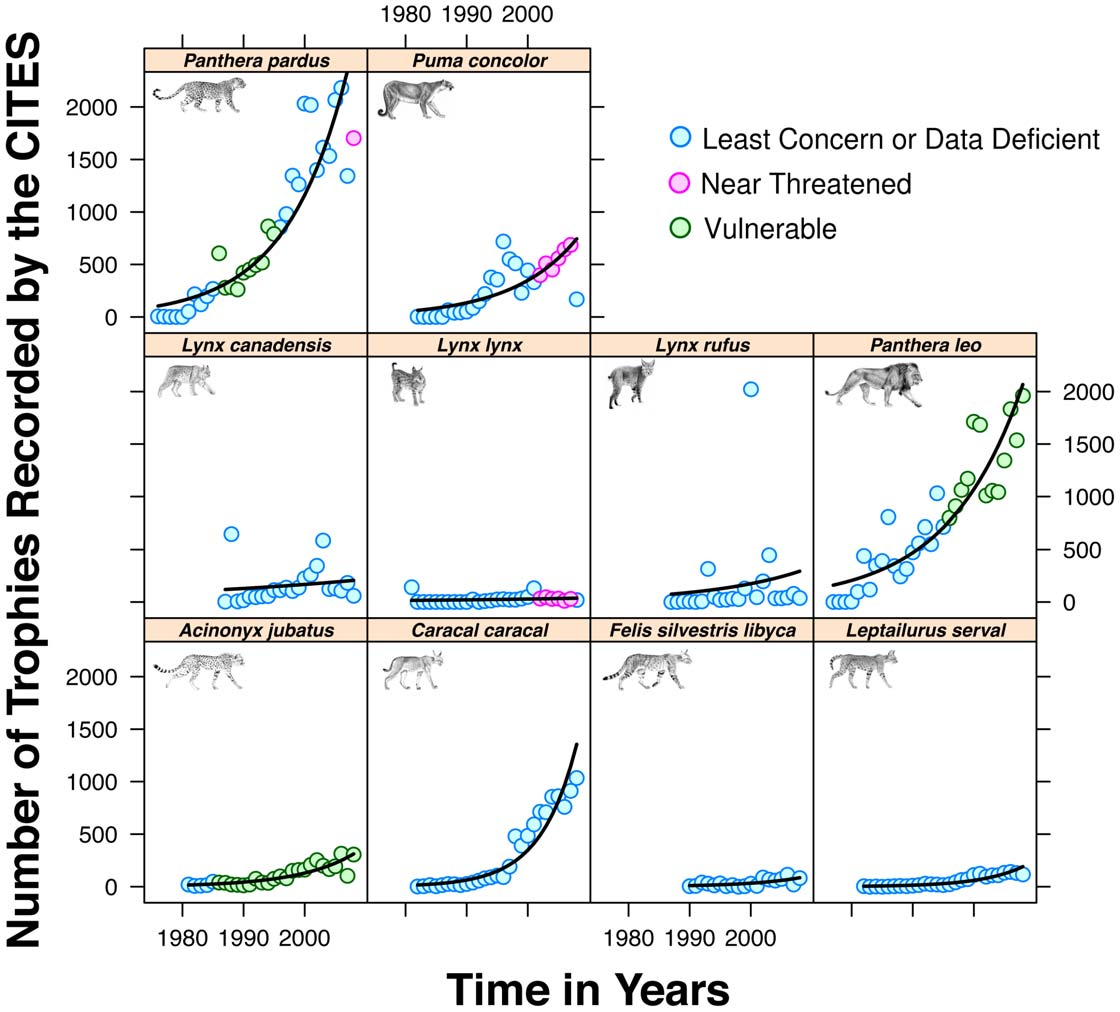
Increase of the number of hunts with time for ten felid species (1975–2010; Poisson regression: c^2^_1_ = 31606; *p*<0.001). IUCN protection status is shown by points of different colours.

We first assessed the intensity of trophy hunting activity by recording the number of legal trophy hunts worldwide over the period from 1975 to 2008, as declared to CITES [15]. We found a marked increase in the number of individuals reportedly killed for seven out of the ten felid species (Figure 1). This increase was exponential for six of them.

Furthermore, we considered the IUCN threat status of hunted felids [15]. Counter-intuitively, the increase of trophy hunting was among the largest for some of the most threatened hunted felids. In particular, numbers of hunts of lions, cheetahs and leopards (all Vulnerable or Near Threatened) doubled every 7.2 years, on average, versus every 11.7 years for the other seven, less threatened felids (Figure 1). Obviously, such an increasing hunting pressure can eventually have dramatic consequences on the populations.

**Figure 2:**
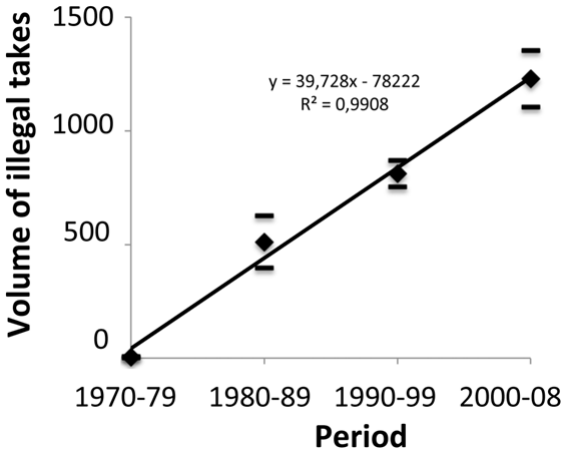
Changes in time of the volume of illegal trade of felid species between 1975 and 2010, as recorded by the CITES Databases.

Thirdly, we analysed the volume of illegal takes for felids from the CITES database (including only “trophies”, “skins” and “skulls”) and show that during the study period the illegal takes have been increasing linearly (Figure 2). Wildlife trade is now recognized a major commercial activity of transnational organized crime [16], [17] and it is unlikely that this dramatic increase could be solely due to an exponential efficiency of custom controls. Customs are obviously increasingly aware and efficient but so are illegal wildlife traders and it is believed that increased quantities of seizures in customs also reflect the intensification of this multi-billion dollar illegal industry [16]. It is indeed likely that custom efficiency increase ought to be accompanied (or preceded) by a similar efficiency increase of smuggler's methods and networks (akin to the Red Queen paradigm, van Valen, 1973). Figure 2 shows that illegal trade too is increasing. Meanwhile, according to the IUCN, the populations of all four species of conservation concern and one Least Concerned species are currently declining [18]. The increasing vulnerability (and associated decreasing availability) of species is not an impediment to increased hunting pressure.

**Figure 3:**
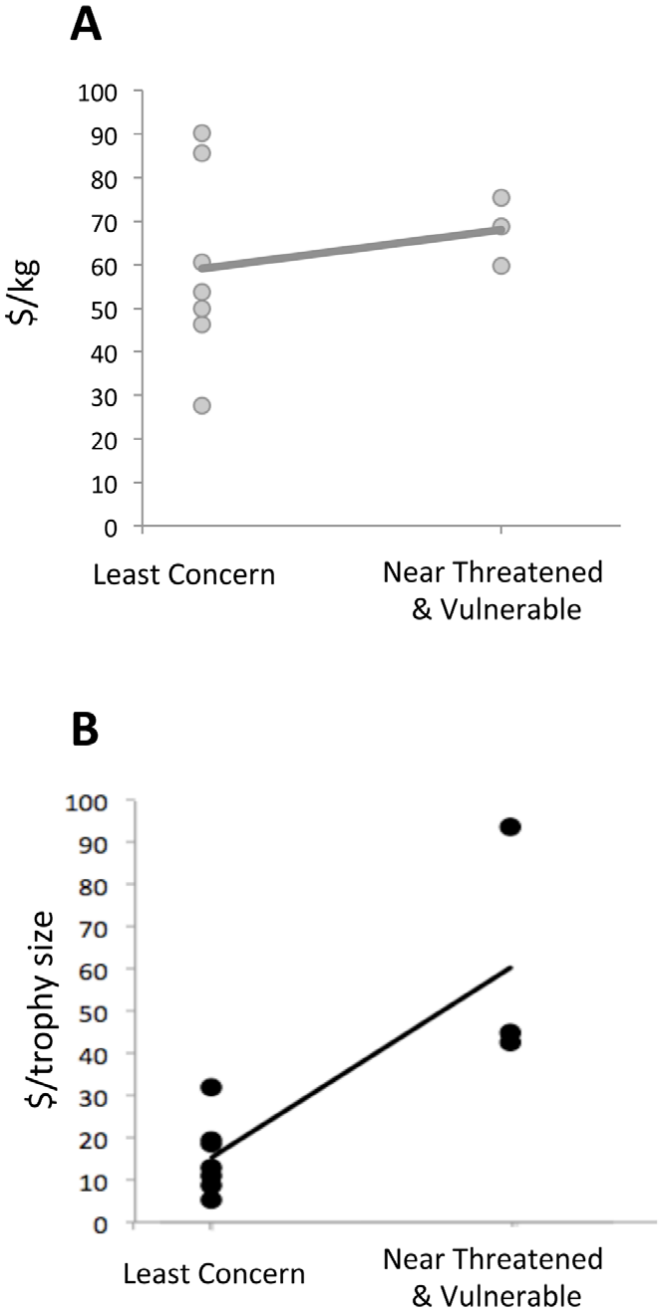
Positive relationship between the IUCN protection status and the price. Trophy price has previously been corrected by body mass, in kg, grey dots (Kruskal test: A : H_1_ = 0.64; *p* = 0.42), or by trophy size, in SCI index, black dots (B: H_1_ = 5.72; *p* = 0.02).

Next, we analyzed the monetary value (trophy hunting fee) of each species and found that Near Threatened and Vulnerable species are more valued, regardless of their body mass or trophy size (Kruskal test: (A) H_1_ = 0.63; *p* = 0.42; (B) H_1_ = 5.72; *p* = 0.02, Figure 3).

**Figure 4:**
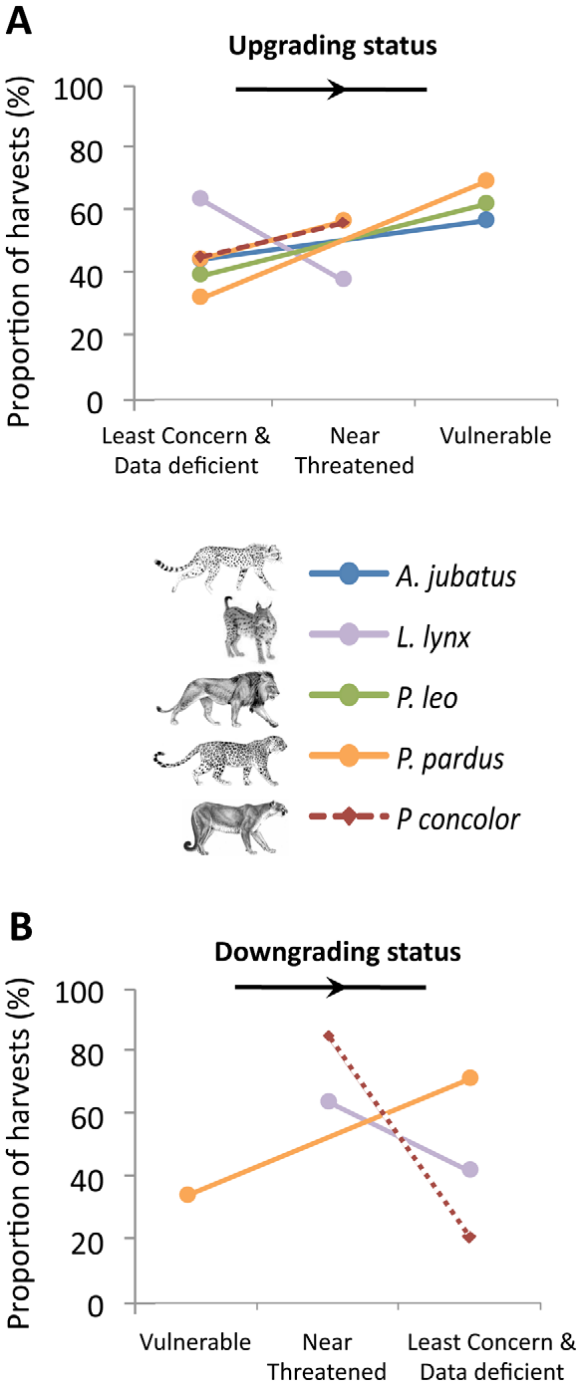
Trend of the number of hunts following an IUCN status change. Status changes, that can be an increased (A) or a decreased (B) IUCN protection status, show that protection status is directly related to attractiveness and exploitation (marginal significance, A: R^2^ = 0.87; F_2,44_ = 2.81; *p* = 0.070; B: R^2^ = 0.85; F_2,9_ = 3.91; *p* = 0.060). Note that *P. pardus* experienced several successive status changes.

Last, we analyzed the effect of changes in IUCN threat status on the volume of hunts. Unexpectedly, we found that declaring a species more threatened has perverse conservation consequences. Indeed, upgrading the species from Least Concern to Near Threatened led to some increases of trophy numbers, while upgrading to Vulnerable, a higher threat status, led to an even more marked increase (R^2^ = 0.87; F_2,44_ = 2.81; *p* = 0.070, Figure 4A). Most surprisingly, a status downgrade led to some decrease of species exploitation (R^2^ = 0.85; F_2,9_ = 3.91; *p* = 0.060, Figure 4B), thereby suggesting a consecutive reduction of their attractiveness to hunters. This analysis strongly suggests a causal relationship between threat status and volumes hunted. However, it does not show merely that hunting makes felid species vulnerable to extinction, but rather, surprisingly, that vulnerability to extinction makes felid species more hunted.

## Discussion

In this paper, we have shown that trophy hunting could constitute an underestimated threat to fragile felid species since the value of rarity makes them disproportionately desirable. With the ten felids species currently hunted for their trophies, we demonstrated that the number of killed individuals increases with time, in several cases exponentially, despite established populations declines. We also show that the price of trophies is dependent on species threat status and that changes in threat status result in counter-intuitive changes in hunting pressures. Indeed, our results indicate that an increase in species threat status coincides with increasing hunting effort, while a downgrading to a lower threat status paradoxically results in a reduction in hunting pressure. Together, this suggests a possible over-exploitation of trophy-hunted felids and the urgent necessity of a better, scientifically quantified quota system coupled with reconsidered protection methods.

Rarer species are generally more attractive and of higher value than similar common species [14]. Experiments in zoos and web-based questionnaires have demonstrated the value of rarity for plant and animal species [19], [20]. The disproportionate value and resulting exploitation of rare species has been evidenced in markets as different as exotic pet collections [21], luxury good consumption [22] and ecotourism [23]. In addition, the relationship we show here for felids has also been demonstrated for trophy hunting of ungulates [13], [24].

Motivations for trophy hunting may be various [3] but typically hunters' target selection is driven by the challenge of the hunt, with the most difficult species to hunt being the most rewarding. With technological progresses, such as firearms, motor vehicles and other modern practicalities, it has been advocated that the challenge of hunting has shifted from the perilous or difficult to hunt towards the rare species [14]. Indeed, one very likely explanation for the desirability of threatened species is their associated rarity rather than their vulnerability *per se*, as many hunters advocate their commitment to conservation. Because of limited supply, greater wealth or power is necessary to acquire one of the very few permits delivered for the least abundant species. The successful hunter wins a competition for restricted goods and gains prestige among his peers [14], [23]. It is likely that the ever-increasing mismatch between attractiveness and availability also stimulates illegal hunting. Our analyses show that illegal harvest, by definition difficult to quantify, constitutes an underappreciated threat to felid species that are subject to stringent hunting quotas, if these restrictions are not efficiently enforced.

Although it is arguably difficult to unambiguously attribute causation to a correlation, the breath of our argument, together with the demonstrated effect of rarity value in different markets, including on ungulates trophy hunting [13], [24] should be sufficient to raise caution about the hidden consequences of trophy hunting on threatened species. Adequately calculated quotas require precise population size assessment, as well as growth capacity and knowledge of density dependent mechanisms (such as Allee effects, [25]), so that harvest can be calculated to be sustainable. One of the strongest arguments of the opponents of trophy hunting is the difficulty to provide and enforce adequate hunting quotas. For example, it is known that in Zimbabwe, trophy licences for lions exceeded the entire population for many years, partly because the population size was unknown [26]. The few felid species for which estimates of total population size are available have dramatically declined from their historical abundance. For example, only 23 000 to 39 000 lions and 7 500 cheetahs now remain from numbers which may have been one order of magnitude higher or more (IUCN Red List 2010). Solid and precise estimate are lacking for most other hunted felids, yet they are all increasingly hunted (Figure 1).

Recent studies have shown that, contrary to claims of hunting proponents, trophy hunting was the main driver of population decline in African lions and leopards [12], [27]. Cases of reduced lion quotas are said to have led to increased prices, up to one order of magnitude during national hunting bans [13], [28]. For lions, the increase in trophies may in part be due to the recent amplification of the practice of “canned lions” (lions that have been raised in reserves for hunting purposes). Yet, wild lions are also disproportionately hunted and lion trophy hunting is, in many cases, unsustainable [26]. We demonstrate here that this attraction to rare species might affect several felids similarly and, outside this family, other species hunted for their trophies could also be affected [13], [14]. In this regard, recent interests in opening trophy hunting of tigers, as the “most expensive trophy in the world” [29] raises new concerns.

Trophy hunting has a unique status in conservation; its benefits have been demonstrated in several cases, where species might even have been saved from extinction by a thorough management program involving harvest of a few selected individuals, protection of the rest of the population, and injection of very significant funds for species and habitat protection [1], [2], [3], [4]. However, harvest based on improperly calculated or enforced quotas may have led to overexploitation of other species. This is especially the case when the attraction for rarity artificially increases trophy value and risks driving them into an extinction vortex [14], [24]. Consequently, if not strictly regulated by quotas that are scientifically established, seriously enforced and internationally organized, the continuous increase of kills in threatened species could risk driving them towards extinction. These considerations are of crucial significance if trophy hunting is to be used appropriately as a conservation tool.

## Materials and Methods

### Data collection

The Safari Club International (SCI) database (available on the web http://www.scirecordbook.org/) was used to obtain the list of hunted species in Felids. Thirteen felid species are subjected to trophy hunting. From the SCI database, we extracted trophy scores (corresponding to trophy size) for each harvest given in points. Male body masses were found in the CRC handbook of mammalian body masses [30]. From the IUCN red list of threatened species, we collected the category under which the 13 felid species were classified annually since 1975. We could therefore identify which species experienced a change in its IUCN status during this period. Caracal (*Caracal caracal*), African wild cat (*Felis silvestris libyca*), Serval (*Leptailurus serval*), Canada Lynx (*Lynx canadensis*) and Bobcat (*Lynx rufus*) were classified as Least Concern during the entire study period. The status of Cheetah (*Acinonyx jubatus*) and African lion (*Panthera leo*) changed once. The status of European lynx (*Lynx lynx*) and Cougar (*Puma concolor*) changed twice. Lastly, the status of leopards (*Panthera pardus*) changed three times. According to the IUCN, the populations of six species are currently declining.

The CITES database on species trade (CITES Trade Database, UNEP World Conservation Monitoring Centre, Cambridge, UK) compiles the data on the international trade of each member countries or parties (actually 175 countries). These correspond to a proxy of the yearly number of legal importation and exportation of trophies reported for each species by the parties from around 1975 to 2008. For each record the corresponding number of individuals was provided. Among the 13 felid species harvested, we excluded tigers (*Panthera tigris*), jaguars (*Panthera onca*) and African golden cats (*Profelis aurata*) because we found no harvest the last 5 years in the CITES data base and no trophy price for these species. We did not consider the number of records in 2009 because of the required delay to collect and centralize the information by the CITES. Data on illegal trade were also collected from the CITES database and all items were considered. For items listed as “derivative”, which could be numbering in the thousands likely parts of animals, we considered only one item per record. Although the quality of data collection and reportage probably increased over time, it ought to be globally similar for all species. As we used the number of harvests corrected for the increase with time, the observed increased number of hunts should not be due to such improved quality.

We used the trophy prices reported by Booth (2009) for African felids and collected the trophy fees proposed by different hunting societies on the web for the non-African ones [31]. Two to eight different trophy fees were used to calculate the average price. All prices used run between 2008 and 2010. Trophy fees include the price of one trophy for one species. It does not include any logistic costs for the hunt and is therefore comparable among countries. Instances where the trophy fee could not be distinguished from the other costs of the hunting safari were not considered. Typically, trophy fees are first determined by governments, and then bought by hunting societies that intend to sell them to their clients after increasing the price [31]. In this context, a high price attributed to one trophy by hunting societies means that this type of trophy is highly valued among their clients. It is largely recognized that prices reflect desirability and that the prices increase with the demand [32].

### Statistical Analyzes

We used a Poisson regression to analyze the temporal trend in the annual number of trophies. We first fit a linear trend on the log-scale (equivalent to an exponential model on the normal-scale) to test for an overall trend over time in the number of trophies in Felids. We then tested for the effect of rarity, entered as a two-category factor (Least Concern vs. Near Threatened/Vulnerable), on the rate of increase of the number of trophies listed in the CITES with time by fitting the first order interaction between time and rarity.

Next, we calculated the trophy price per unit (*i.e.*, trophy point and kilo). We used the second biggest trophy score listed in the record book of the Safari Club International in order to avoid any mistake and bias in the reporting, which are more likely in the biggest. This score is determined using the SCI Official Measurer's Manual, a key reference in the field [33]. Thus we used it as a proxy of the trophy size for a given species. We then divided the trophy price by this score to obtain the trophy price per point for each species. In the same way, we calculated the trophy price per body mass kilo. We used the IUCN threat status of the species in 2008 for the comparison between trophy prices. Indeed no status change occurred after 2008 for the present species and the prices used ran between 2008 and 2010. We considered two groups of species because of the low number of data: no protection need (Least Concern, 7 species) versus others (Near Threatened or Vulnerable, 3 species). We then tested for between-group differences in the trophy price per unit (kg or SCI points). The dataset was too small to allow the use of a simple linear model. Hence, we used a Kruskal test to compare trophy prices per units between our samples of rare and common species [34].

To examine the effect of threat status changes on the trade, we calculated the mean number of harvested individuals 5 years before and 5 years after a change in the IUCN protection status of the species, except for *Puma concolor, Lynx lynx and Panthera pardus* whose status was changed too recently to be able to conduct this analysis (*i.e.*, in 2008). In this case, the comparison was performed between the year preceding and the year following the change. The period of 5 years was chosen to smoothen for yearly variations. We used the de-trended number of trophies as the response variable, calculated by modelling the residuals of the Poisson regression linking annual numbers of trophies and time. Therefore, we controlled for the observed general increase of harvest with time. We removed the difference in the global number of harvests among species by using the proportion of harvests instead of the row number of harvests. In this regard, we considered as 100% the sum of the mean harvests before and after the status change. We then tested for the effect of rarity on this parameter. The results obtained using a buffer of 1, 2 or 5 years before and after a change in IUCN status were qualitatively similar.
